# Comparative Analysis of Serum and Tissue miRNA Expression Profiles and Regulatory Pathways in Early-Stage Ovarian Cancer Using Public Databases

**DOI:** 10.3390/ijms27125629

**Published:** 2026-06-22

**Authors:** Shuya Cai, Hui Tan, Xiaoyu Niu, Nirupal Eskar, Zaoling Liu

**Affiliations:** Department of Epidemiology and Health Statistics, School of Public Health, Xinjiang Medical University, Urumqi 830011, China

**Keywords:** early-stage ovarian cancer, MicroRNA, serum, tissue, regulatory pathways, public database

## Abstract

To characterize the distinct expression profiles of microRNAs (miRNAs) in serum and tissue and to delineate the heterogeneity of their regulatory mechanisms in early-stage ovarian cancer (EOC), thereby identifying candidate biomarkers for non-invasive early diagnosis. Differentially expressed miRNAs were identified by integrating publicly available datasets of EOC tissues and serum samples from the Gene Expression Omnibus (GEO) and The Cancer Genome Atlas (TCGA). Core miRNAs were subsequently screened through integrated differential expression analysis, weighted gene co-expression network analysis (WGCNA), and feature importance ranking derived from optimized machine learning models. Protein–protein interaction (PPI) networks and functional enrichment analyses (GO and KEGG) were performed on predicted target genes to systematically compare the functional discrepancies between serum- and tissue-derived miRNAs. No overlapping core miRNAs were observed between the two compartments. Serum miRNAs exhibited an overall up-regulated trend, whereas tissue miRNAs were predominantly down-regulated. Although the regulatory pathways demonstrated significant heterogeneity, they ultimately converged on the cell cycle and the PI3K-Akt signaling pathway, indicating high functional homology. Furthermore, serum miRNAs are not merely passive leakage products from tissues; current evidence suggests they may be selectively packaged into exosomes to participate in tumor regulation. Despite divergent expression profiles, serum and tissue miRNAs share homologous regulatory functions in EOC. These findings suggest that serum miRNAs accurately reflect the core molecular status of tumor tissues, providing a robust molecular foundation for liquid biopsy-based early detection strategies.

## 1. Introduction

Ovarian cancer (OC) ranks among the three most lethal malignancies threatening women’s health and is widely recognized as the most aggressive gynecological neoplasm. Its insidious onset and rapid progression present formidable challenges to clinical management [1]. According to the latest GLOBOCAN estimates from the World Health Organization/International Agency for Research on Cancer (WHO/IARC) in 2024, approximately 324,000 new cases and 207,000 deaths were reported worldwide in 2022, highlighting its substantial global burden [1]. A critical obstacle in combating this disease is its asymptomatic nature during early stages; consequently, nearly 70% of patients are diagnosed with advanced-stage disease [2]. This late-stage diagnosis starkly contrasts with survival outcomes. While the 5-year relative survival rate for advanced OC remains approximately 49%—often complicated by recurrence and chemoresistance—early-stage (FIGO I–II) patients exhibit significantly improved survival rates exceeding 80–90% [2,3]. These striking disparities underscore the pivotal role of early detection in mitigating mortality and improving prognosis.

Current diagnostic modalities for OC primarily rely on imaging techniques and serum tumor biomarker assays [4]; however, both approaches are encumbered by significant limitations. While ultrasonography offers convenience, it is frequently associated with suboptimal sensitivity in early-stage detection [5]. Computed tomography (CT) exhibits high specificity but limited sensitivity [6]; moreover, although magnetic resonance imaging (MRI) provides exquisite anatomical detail [7], its prohibitive cost precludes its application in population-wide screening. Compounding these issues, the serum biomarker carbohydrate antigen 125 (CA125) lacks sufficient sensitivity and specificity for early-stage disease. Furthermore, its levels can be elevated in benign gynecological conditions, such as endometriosis and pelvic inflammatory disease, leading to unacceptably high false-positive rates [8]. Similarly, although human epididymis protein 4 (HE4) demonstrates adjunctive utility in differentiating benign from malignant pelvic masses, its standalone sensitivity for early screening remains unsatisfactory [9]. Consequently, there is an urgent clinical imperative to identify novel diagnostic biomarkers that combine high sensitivity and specificity with the capacity for non-invasive dynamic monitoring. Among various liquid biopsy candidates, microRNAs (miRNAs) have emerged as particularly promising. As endogenous non-coding RNAs, miRNAs orchestrate pivotal roles in critical biological processes—including tumor proliferation and differentiation—via post-transcriptional regulation of target genes [10]. Crucially, their encapsulation within extracellular vesicles or protein complexes confers remarkable stability in circulation, enabling them to mirror the dynamic molecular landscape of tumors in real time. This attribute, coupled with straightforward detection protocols and relatively low costs, renders miRNAs highly amenable to large-scale clinical translation [11,12].

However, significant gaps remain in miRNA research concerning early-stage OC (FIGO I–II). First, a pronounced disparity in sample type utilization persists. While the majority of studies focus on tumor tissues [13]—whose miRNA profiles, though invasive to acquire, are regarded as the gold standard for deciphering in situ tumorigenic mechanisms [14]—research on circulating miRNAs in serum remains comparatively scarce. Moreover, existing studies largely treat serum and tissue as discrete entities, lacking systematic comparative analyses to correlate the key pathways enriched in circulating miRNAs with those in tumor tissues. Consequently, it remains unclear whether the pathways enriched in serum are homologous to the core pathways driving tumorigenesis or if serum signatures can genuinely recapitulate the molecular essence of the tumor. On the other hand, notable limitations exist in the algorithmic selection for diagnostic model construction. Current research on miRNA-based ovarian cancer diagnostics predominantly relies on single machine learning algorithms for modeling analysis. For instance, some studies have constructed classification models solely based on support vector machines (SVM) [15], while others have utilized LASSO regression alone to build predictive models [16] or employed neural networks to evaluate prognostic factors [17], without conducting horizontal comparisons or comprehensive evaluations across multiple algorithms. Models built on a single algorithm are susceptible to the algorithm’s inherent biases and the specific characteristics of data distribution, hindering an objective assessment of their true efficacy. This limitation ultimately results in insufficient generalization capability when models are validated against external independent cohorts.

Tumor initiation and progression are contingent upon the coordinated dysregulation of multiple functionally interrelated miRNAs [18]. Conventional screening methods—such as differential expression analysis, LASSO, and SVM—focus exclusively on individual molecular expression profiles, failing to capture synergistic interactions among miRNAs. Consequently, these approaches are prone to overlooking pivotal molecules that exhibit subtle expression changes yet occupy central positions within regulatory networks, thereby yielding results of limited biological significance. Weighted gene co-expression network analysis (WGCNA), a systems biology methodology, addresses this limitation by aggregating co-expressed miRNAs into modules and identifying core modules and hub miRNAs relative to clinical traits. By circumventing arbitrary cut-off thresholds, this approach leverages network topology to identify low-abundance yet pivotal miRNAs, aligning with the polygenic pathogenesis characteristic of early-stage ovarian cancer. This provides a methodological foundation for mining functional miRNA modules from both serum and tissue specimens in the present study. Previous studies have substantiated the efficacy of WGCNA in biomarker and therapeutic target discovery across diverse diseases, including gynecological malignancies [19,20].

In summary, despite the pressing clinical need for early detection, the management of OC remains constrained by the limitations of current diagnostic modalities. While miRNAs hold considerable promise as biomarkers, existing studies are often compromised by sample type imbalances, algorithmic homogeneity, and a paucity of insight into inter-miRNA synergy. To bridge these critical gaps, the present study leverages publicly available serum and tissue miRNA datasets to conduct a comprehensive analysis of early-stage OC. We integrated differential expression analysis with weighted gene co-expression network analysis (WGCNA) to identify core miRNAs and subsequently constructed and validated diagnostic models using multiple machine learning algorithms to ensure robustness. Furthermore, we performed target gene prediction and pathway enrichment analyses to systematically compare the core regulatory pathways and biological functions between serum and tissue compartments. The primary objective was to elucidate the functional concordance between circulating cell-free miRNAs and tissue-resident miRNAs, specifically determining whether serum-derived regulatory networks can authentically recapitulate the molecular hallmarks of tumorigenesis. Ultimately, this study aims to substantiate the theoretical foundation for developing blood-based, non-invasive early detection strategies for ovarian cancer.

## 2. Results

### 2.1. Baseline Demographic Characteristics

Comparative analysis of patient age across the serum and tissue cohorts revealed distinct patterns. In the serum analysis cohort (*n* = 110), patients with Stage II disease were significantly older than those with Stage I (*p* = 0.002). Analogous trends toward older age in Stage II patients were observed in both the serum external validation cohort (*n* = 168) and the tissue analysis cohort (*n* = 87), with differences reaching statistical significance (*p* = 0.039 and *p* = 0.039, respectively). Conversely, no statistically significant age difference was detected between stages in the tissue external validation cohort (*n* = 34) (*p* = 0.397); this lack of significance is likely attributable to the limited sample size, which may have been underpowered to detect genuine differences in age distribution. Detailed demographic characteristics are summarized in Table 1.

Owing to the inherent constraints of publicly available datasets, the GEO datasets utilized in this study provided only de-identified age and tumor stage information. In contrast, the TCGA cohort additionally included pathological data such as menopausal status, treatment history, and survival outcomes. Given the heterogeneity in clinical indicator collection across cohorts, only baseline data amenable to uniform comparison are presented in Table 1.

### 2.2. Results of Differential Expression Analysis in Serum and Tissues

To ensure the robustness and reliability of the differentially expressed miRNA (DE-miRNA) screening, three mainstream algorithms—limma, edgeR, and DESeq2—were independently implemented; the intersected results from all three were adopted as the candidate DE-miRNA set.

Serum differential expression analysis yielded 1003 DE-miRNAs (841 upregulated and 162 downregulated) following the intersection of results from the three algorithms. In contrast, tissue differential expression analysis identified 231 DE-miRNAs (117 upregulated and 114 downregulated) after intersection.

**Figure 1:**
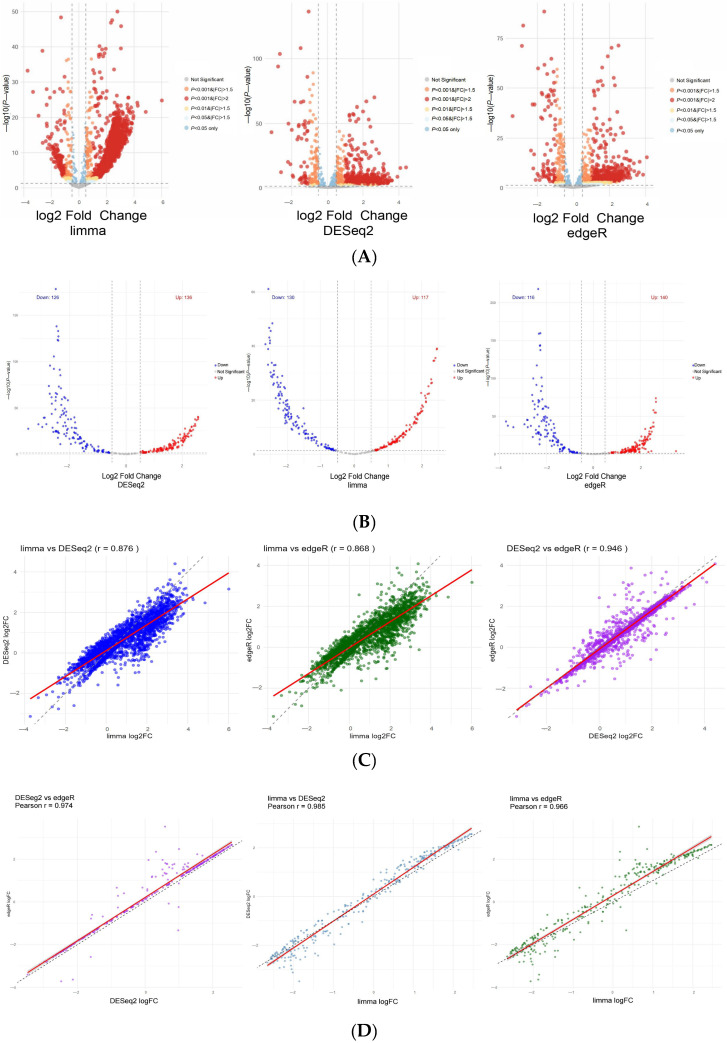
Volcano plot of differentially expressed miRNAs and consistency analysis between methods. Differential expression analysis of miRNAs in the training sets of the serum and tissue cohorts, derived from the Gene Expression Omnibus (GEO) database. The criteria for differential expression were set at |log_2_FC| > 0.5 and *p* < 0.05. (**A**) Volcano plots illustrating differentially expressed miRNAs in the serum cohort. The x-axis represents the log_2_ fold change (log_2_FC), and the y-axis represents −log_10_(*p*). Results from three algorithms—limma, DESeq2, and edgeR—are displayed. The horizontal dashed line corresponds to a *p*-value threshold of 0.05, and the vertical dashed lines are set at log2FC = ±0.585, representing fold change values of 1.5 and 0.667, respectively. (**B**) Volcano plots illustrating differentially expressed miRNAs in the tissue cohort. Axis definitions are consistent with those in Figure 1A, presenting results from DESeq2, limma, and edgeR sequentially. (**C**) Pairwise scatter plots comparing log_2_FC values in the serum cohort. The x- and y-axes represent log_2_FC values calculated by two different algorithms, visually assessing the concordance between them. (**D**) Pairwise scatter plots comparing log_2_FC values in the tissue cohort. Axis definitions are consistent with those in Figure 1C, displaying the correspondence of log_2_FC under pairwise combinations of the three algorithms. To enhance the reliability of feature selection, differentially expressed miRNAs identified by all three algorithms were intersected. All subsequent downstream analyses were uniformly based on the output results from the limma package.

Figure 1A,B illustrate the volcano plots from the three algorithms for both serum and tissue cohorts, clearly delineating the distribution of miRNAs across varying significance levels and fold-change magnitudes. Furthermore, the pairwise log_2_FC scatter plots in Figure 1C,D demonstrate that the Pearson correlation coefficients among the three algorithms all exceeded 0.86, indicating a high degree of concordance among limma, edgeR, and DESeq2. This corroborates the stability and reliability of the differential expression screening process.

### 2.3. WGCNA and Hub miRNA Screening in Serum and Tissues

**Figure 2:**
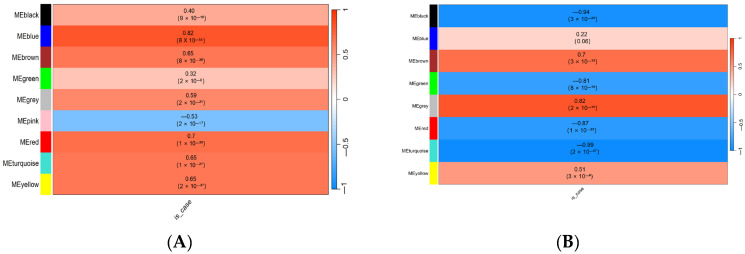
WGCNA modules—phenotypic association analysis of serum and tissue cohorts. This figure was generated using data from the respective training sets. (**A**) Heatmap illustrating the correlation between module eigengenes and phenotypes in the serum cohort. (**B**) Heatmap illustrating the correlation between module eigengenes and phenotypes in the tissue cohort. The values within the heatmaps represent the Pearson correlation coefficients between the module eigengenes and early-stage ovarian cancer status, with corresponding *p*-values indicated in parentheses. Color intensity reflects the strength of the correlation.

For serum samples, a signed weighted co-expression network was constructed. A soft-thresholding power range of 1–30 was explored, and the optimal parameter was selected based on the scale-free topology fit index (signed R^2^) and mean connectivity. A power of 23 was identified as optimal, yielding a signed R^2^ of 0.813 (meeting the criterion of R^2^ ≥ 0.8) and a mean connectivity of 1.0. This parameter balanced the scale-free property with structural stability, effectively circumventing module fragmentation associated with higher power values. Module identification was performed using uniform parameters (deepSplit = 2, pamRespectsDendro = FALSE, minClusterSize = 30), resulting in nine co-expression modules. Correlation analysis between module eigengenes and phenotypes revealed that the MEblue module was significantly correlated with early-stage ovarian cancer status (*p* < 0.001; Figure 2A), from which 38 key miRNAs were extracted. Intersecting these WGCNA-identified key miRNAs with serum DE-miRNAs and applying the natural breaks (Jenks) method for Gene Significance (GS) yielded eight final serum core miRNAs.

Similarly, for tissue samples, a signed weighted co-expression network was established. The optimal soft-thresholding power was set at 17 (signed R^2^ = 0.802), satisfying the scale-free network fit criterion. Network construction parameters were set as blockSize = min(2000, ncol(datExpr)) and verbose = 5, while module detection parameters were kept consistent with those used for the serum cohort, ultimately generating eight co-expression modules. The MEturquoise module exhibited a significant correlation with the early-stage ovarian cancer phenotype (*p* < 0.001; Figure 2B). Applying the same GS and MM thresholds yielded 56 key miRNAs. Subsequent intersection filtering and natural breaks analysis identified eight final tissue core miRNAs. Detailed information is provided in Table 2.

### 2.4. Performance Comparison of Machine Learning Models in Serum and Tissues

**Figure 3:**
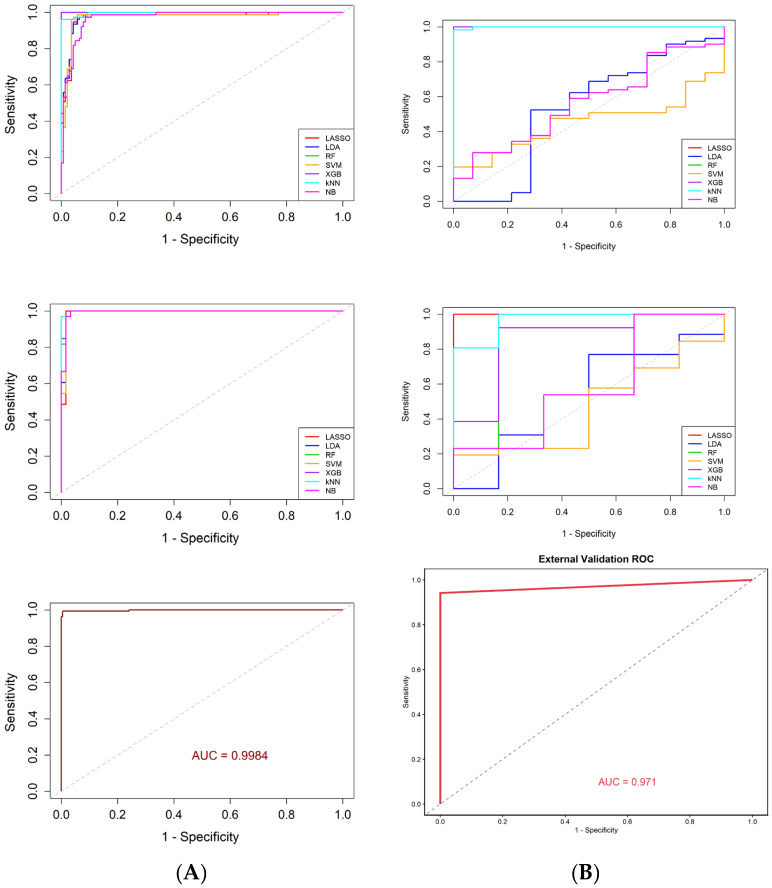

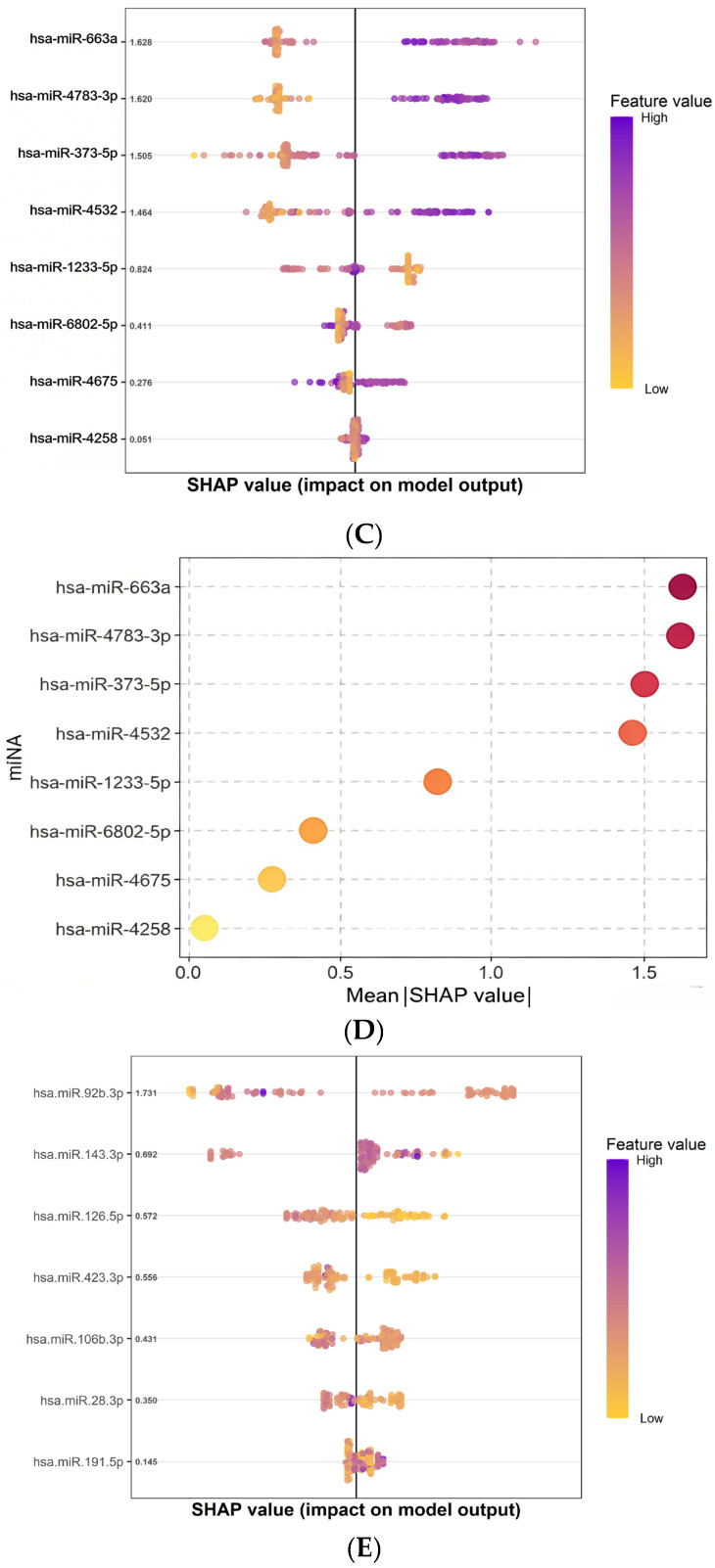

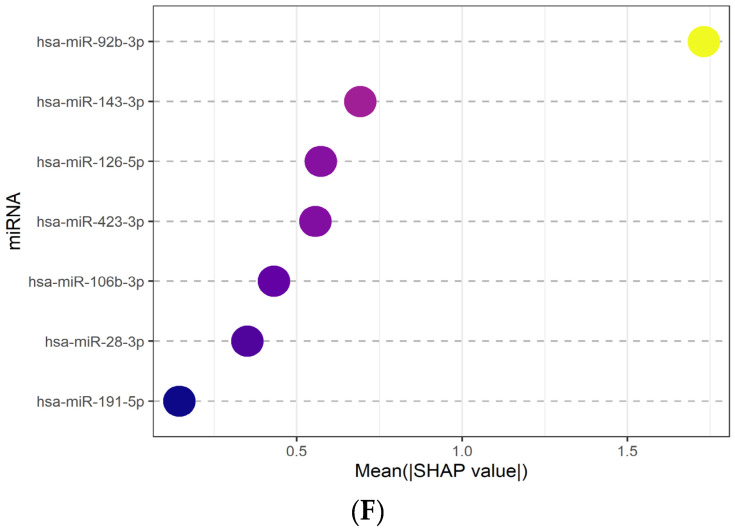
Performance evaluation and interpretability analysis of the diagnostic model. Leveraging the identified core miRNAs, seven machine learning classifiers were constructed: Least Absolute Shrinkage and Selection Operator (LASSO), Linear Discriminant Analysis (LDA), Random Forest (RF), Support Vector Machine (SVM), Extreme Gradient Boosting (XGBoost), k-Nearest Neighbors (kNN), and Naive Bayes (NB). (**A**) Receiver Operating Characteristic (ROC) curves for the serum cohort models. From top to bottom, the panels depict model performance across the training, internal validation, and external validation sets. (**B**) ROC curves for the tissue cohort models, arranged identically to the serum cohort. The x-axis represents 1 − specificity, and the y-axis represents sensitivity. The Area Under the Curve (AUC) served as the primary metric for evaluating diagnostic performance. (**C**) SHAP (SHapley Additive exPlanations) swarm plot for the optimal serum model (XGBoost), visualizing the magnitude and direction of each core miRNA’s contribution to the model output. (**D**) Feature importance ranking plot for the optimal serum model, quantitatively delineating the weight distribution of each core miRNA in the classification task. For panel (**D**), the color gradient of the points ranges from light yellow to deep red. The warmer red tones correspond to higher average absolute SHAP values and greater diagnostic contributions of miRNA features. (**E**) SHAP swarm plot for the tissue model (XGBoost), reflecting the contribution patterns of core miRNAs to the tissue model predictions. (**F**) Feature importance ranking plot for the tissue model (XGBoost), delineating the hierarchical importance of each core miRNA in the tissue diagnostic model. In Figure (**F**), the color of the dots gradually transitions from dark blue to purple, and then to bright yellow; the bright yellow dots represent miRNAs with larger average absolute SHAP values and stronger weights in the tissue diagnosis model.

Seven machine learning classifiers were constructed based on the serum core miRNAs: Least Absolute Shrinkage and Selection Operator (LASSO), Linear Discriminant Analysis (LDA), Random Forest (RF), Support Vector Machine (SVM), eXtreme Gradient Boosting (XGBoost), k-Nearest Neighbors (kNN), and Naive Bayes (NB). Key performance metrics for each model across the training, internal validation, and external validation sets are detailed in Supplementary Table S1. Model selection prioritized stability on the internal validation set and generalization performance on the external validation set, rather than relying solely on training set efficacy. In the training set, the RF and XGBoost models achieved perfect AUCs of 1.0, indicating potential overfitting risks, while all other models yielded AUCs exceeding 0.97. In the internal validation set, all models demonstrated stable performance with AUCs > 0.99. Upon comprehensive comparison, the XGBoost model exhibited robust stability during internal validation and superior generalization in the independent external cohort; thus, it was selected as the optimal model. Feature importance analysis identified hsa-miR-663a, hsa-miR-4783-3p, and hsa-miR-373-5p as the top three contributors. Further SHapley Additive exPlanations (SHAP) analysis revealed that hsa-miR-663a, hsa-miR-4783-3p, and hsa-miR-4532 exerted positive contributions to the model output, whereas hsa-miR-1233-5p exerted a negative contribution. ROC curves for all serum models are presented in Figure 3A, with feature importance and SHAP analyses for the XGBoost model shown in Figure 3C,D.

For the tissue cohort, one miRNA absent from the external validation set was excluded, and seven models were constructed using the remaining seven core miRNAs. Performance metrics are detailed in Supplementary Table S1. Consistent with the serum analysis, model selection prioritized validation set stability and generalization capacity over training set AUC. In the training set, kNN and RF performed best (AUCs of 0.994 and 0.998, respectively), while the XGBoost model, despite achieving near-perfect scores, exhibited signs of overfitting and limited generalization. In the internal validation set, kNN (AUC = 0.958), RF (AUC = 0.865), and XGBoost (AUC = 0.859) showed decreased performance compared to their training results, confirming the overfitting tendency of the XGBoost model. Integrating internal and external validation results, the kNN model demonstrated the best overall stability and generalization capability, establishing it as the optimal diagnostic model for the tissue cohort. This advantage was further validated in the external test set. ROC curves for tissue models are shown in Figure 3B. Although kNN achieved the best diagnostic performance, its inherent opacity limits direct interpretation of feature contributions. Therefore, the interpretable XGBoost model was additionally employed for SHAP and feature importance analyses (Figure 3E,F). Feature importance analysis identified hsa-miR-92b-3p, hsa-miR-143-3p, hsa-miR-126-5p, and hsa-miR-423-3p as the most influential miRNAs. SHAP analysis further elucidated their directional impact: high expression of hsa-miR-92b-3p, hsa-miR-126-5p, and hsa-miR-423-3p was negatively associated with ovarian cancer prediction, whereas high expression of hsa-miR-143-3p was positively associated.

### 2.5. Serum and Tissue Target Gene Prediction and Functional Enrichment Analysis

**Figure 4:**
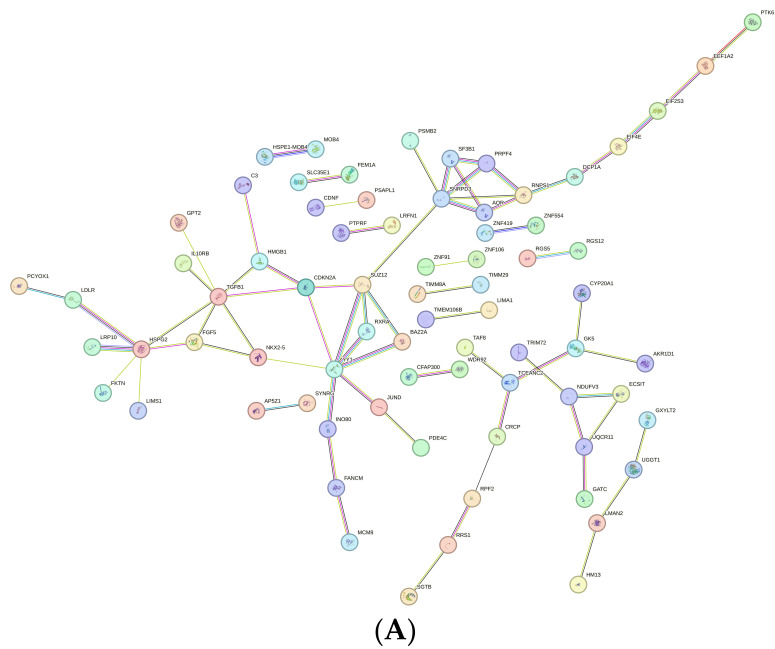

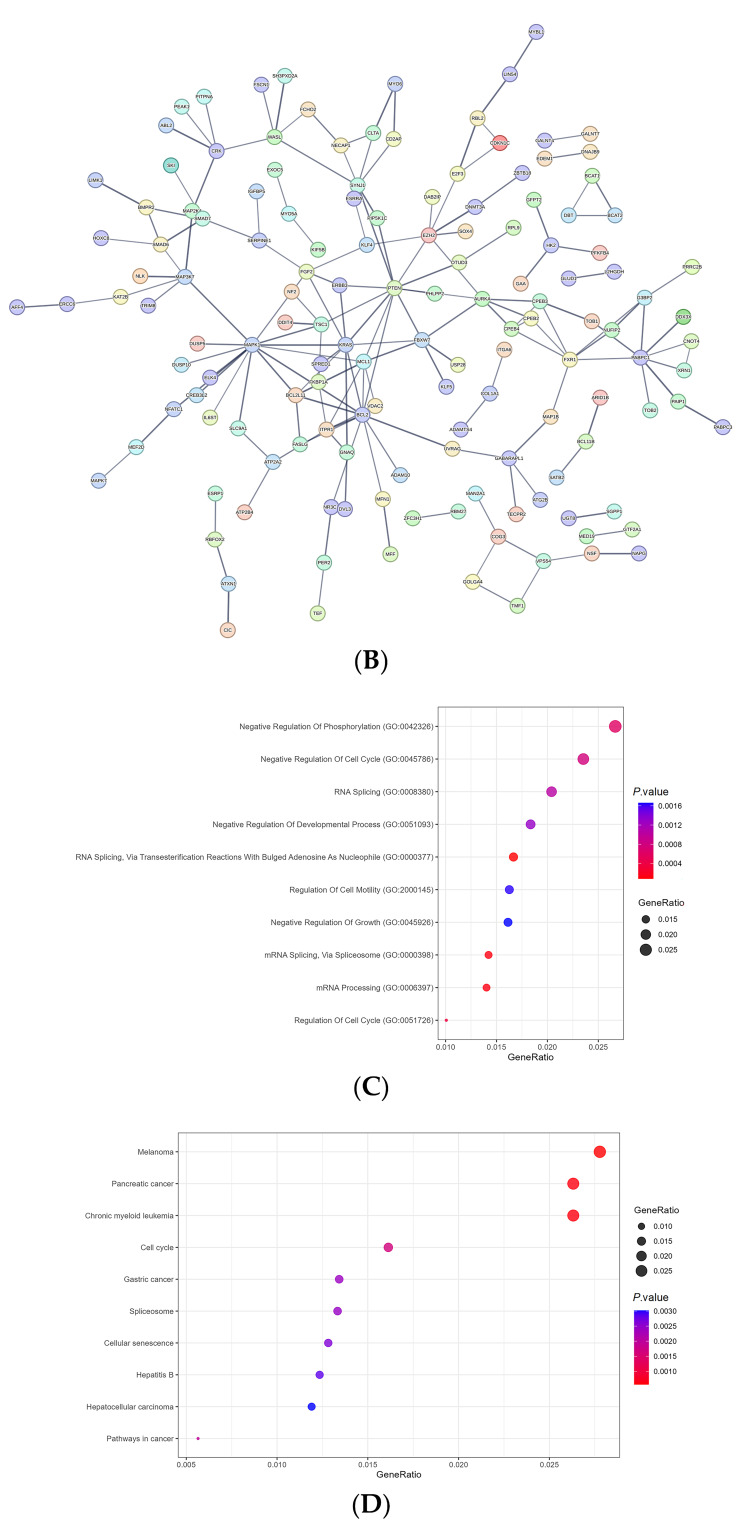

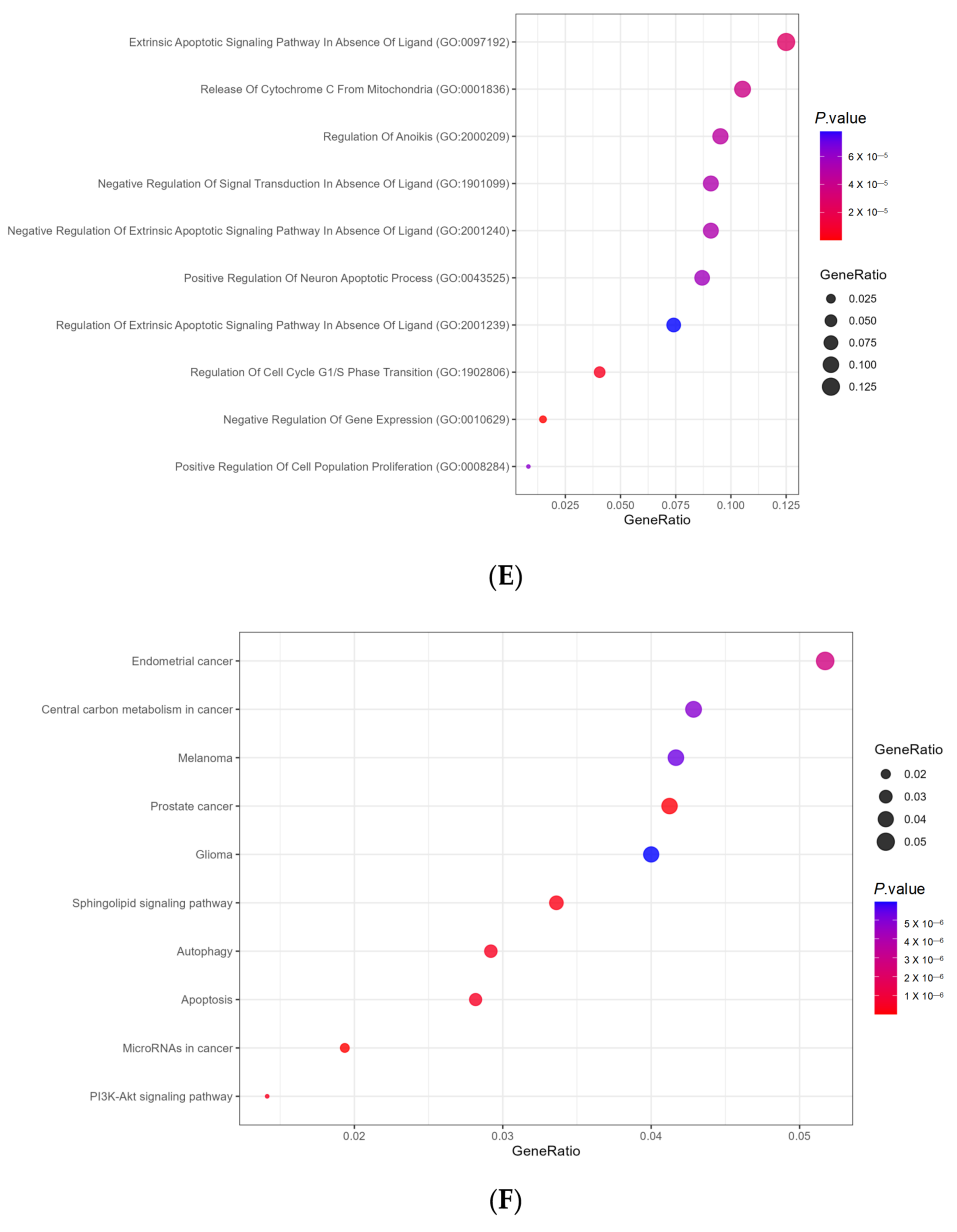
PPI network of miRNA target genes in serum and tissue, and functional enrichment analysis. Target genes of the core miRNAs were collectively predicted using three major databases: TargetScan, miRDB, and miRTarBase. The significance threshold for functional enrichment analysis was set at *p* < 0.05. (**A**) Protein–protein interaction (PPI) network for target genes of the serum cohort core miRNAs. Nodes represent target genes, and edges denote protein–protein interactions. (**B**) PPI network for target genes of the tissue cohort core miRNAs, with graphical elements interpreted analogously to the serum cohort network. (**C**) Gene Ontology Biological Process (GO-BP) enrichment bubble plot for target genes of the serum cohort. (**D**) Kyoto Encyclopedia of Genes and Genomes (KEGG) pathway enrichment bubble plot for target genes of the serum cohort. (**E**) GO-BP enrichment bubble plot for target genes of the tissue cohort. (**F**) KEGG pathway enrichment bubble plot for target genes of the tissue cohort. In all bubble plots, bubble size is proportional to the number of enriched genes, and color intensity corresponds to the statistical significance of the enrichment.

Top-ranked key core miRNAs from both serum and tissue cohorts were selected based on feature importance analysis to predict downstream target genes. Predictions were derived from three major databases, retaining only the intersecting targets, which were then merged and deduplicated. This process yielded 325 intersecting target genes for the tissue core miRNAs and 159 for the serum core miRNAs. Separate protein–protein interaction (PPI) networks were constructed for each gene set, followed by topological analysis to identify the top 10 hub genes ranked by degree value (Figure 4A,B). Functional and pathway enrichment analyses were subsequently performed based on these hub genes.

Enrichment analysis of the top 10 hub genes derived from serum core miRNAs was conducted (Figure 4C,D). KEGG analysis revealed significant enrichment in tumor-related pathways, including melanoma, pancreatic cancer, and the cell cycle, with all pathways meeting the criterion of *p* < 0.05. Gene Ontology biological process (GO-BP) analysis indicated predominant involvement in the negative regulation of phosphorylation, the cell cycle, and RNA splicing. These findings suggest that serum core miRNAs drive the pathogenesis of early-stage ovarian cancer by orchestrating these critical pathways and biological processes.

Enrichment analysis of the top 10 hub genes derived from tissue core miRNAs was also performed (Figure 4E,F). KEGG analysis highlighted enrichment in endometrial cancer, prostate cancer, melanoma, the PI3K-Akt signaling pathway, and apoptosis. GO-BP analysis demonstrated significant involvement in extrinsic apoptotic signaling, the G1/S transition of the mitotic cell cycle, and the regulation of cell proliferation (*p* < 0.05). These results indicate that tissue core miRNAs promote the pathological progression of early-stage ovarian cancer by dysregulating key pathways related to apoptosis, the cell cycle, and the PI3K-Akt signaling cascade.

## 3. Discussion

Early-stage ovarian cancer often evades detection, primarily due to the suboptimal sensitivity of conventional clinical modalities. MicroRNAs (miRNAs) exhibit dysregulated expression profiles within tumor tissues yet possess remarkable stability in bodily fluids, positioning them as promising candidates for non-invasive biomarker discovery. This study conducted a comparative analysis of serum and tissue miRNA profiles in early-stage ovarian cancer. By integrating machine learning modeling, protein–protein interaction (PPI) network analysis, and functional enrichment analysis, we elucidated the distinct expression patterns and regulatory functions of these miRNAs. Our findings delineate the pivotal roles of core miRNAs in the pathological progression of ovarian cancer and establish a theoretical foundation for identifying novel non-invasive diagnostic biomarkers for this malignancy.

Upon identification of the optimal model, SHapley Additive exPlanations (SHAP) values were utilized to quantify the contribution of each core miRNA to the model’s decision-making process. miRNAs with the highest mean absolute SHAP values were selected for preliminary bioinformatics investigation. In the serum model, hsa-miR-663a, hsa-miR-4783-3p, hsa-miR-373-5p, and hsa-miR-4532 were identified as the foremost positive drivers. Notably, miR-663a and miR-373-5p have been experimentally validated to promote oncogenesis, regulate invasion, and serve as promising early diagnostic biomarkers in ovarian cancer [21,22,23]. Conversely, empirical evidence regarding hsa-miR-4783-3p and hsa-miR-4532 in ovarian cancer remains unreported; current understanding is confined to bioinformatics analyses and machine learning predictions derived from public datasets, which indicate their significant upregulation in serum samples from affected individuals [24]. In summary, the biological functions of these miRNAs warrant rigorous experimental validation. The SHAP-based findings presented herein provide preliminary computational insights into their potential clinical relevance, rather than constituting definitive mechanistic evidence.

The core miRNAs identified in the tissue cohort predominantly function as tumor suppressors. Specifically, hsa-miR-92b-3p, hsa-miR-126-5p, and hsa-miR-143-3p have been experimentally validated to modulate ovarian cancer cell proliferation, invasion, and patient prognosis [25,26,27]. While our SHAP analysis identified these miRNAs as positive contributors to the model, it is imperative to clarify that, in a multi-marker panel, a molecule’s contribution reflects its discriminative capacity between groups rather than its intrinsic oncogenic or suppressive function. Thus, this observation does not conflict with previous cellular experimental conclusions. Owing to the inherent limitations of the study design, all functional interpretations presented herein are derived from existing literature and bioinformatics analyses; the precise molecular mechanisms require further validation through in vivo and in vitro experimentation.

A protein–protein interaction (PPI) network was constructed based on the predicted target genes. The results demonstrated that although the target gene sets of the core miRNAs differed between the serum and tissue cohorts, they occupied pivotal topological positions within their respective networks. In the serum PPI network, core nodes—such as YY1 and TGFB1—primarily regulated inflammatory responses, stromal remodeling, and multiple stress signaling pathways [28]. Conversely, core nodes in the tissue PPI network—including MAPK1, BCL2, and PTEN—were extensively involved in the activation of oncogenic signaling, the regulation of apoptosis, and the disruption of tumor suppressor functions [29,30]. Integrating these network characteristics, we propose a preliminary hypothesis: serum- and tissue-derived miRNAs may exhibit functional complementarity. Specifically, tissue miRNAs likely orchestrate malignant regulatory processes within the tumor microenvironment, whereas serum miRNAs may indirectly reflect the systemic pathological dysregulation associated with tumorigenesis. It is important to emphasize that this inference is derived solely from bioinformatics analyses and warrants further validation through subsequent experimental and cohort studies.

Subsequent Gene Ontology (GO) and Kyoto Encyclopedia of Genes and Genomes (KEGG) pathway enrichment analyses were performed on the target gene sets from both cohorts. The results revealed that the regulatory pathways modulated by these miRNAs exhibit both convergence and divergence. Both serum- and tissue-derived miRNAs converged on core pathways governing the cell cycle and tumor apoptosis. This molecular-level consistency substantiates that circulating serum miRNAs mirror the core molecular signatures of tumor tissues, thereby supporting their utility as non-invasive diagnostic biomarkers.

Regarding functional divergence, tissue miRNA target genes were predominantly enriched in canonical oncogenic pathways—specifically PI3K-Akt/mTOR—directly orchestrating the malignant phenotype of tumor cells [31]. Conversely, upregulated serum miRNAs primarily exerted negative regulation over the cell cycle, phosphorylation modifications, and RNA splicing processes [32,33,34]. By suppressing host anti-tumor signals and activating oncogenic kinase cascades, these miRNAs appear to drive tumor progression. Notably, our findings supplement the current understanding by demonstrating that serum miRNAs can contribute to early ovarian carcinogenesis through post-transcriptional regulatory mechanisms, thereby refining the existing mechanistic framework.

The core miRNAs identified in serum and tissue exhibited no overlap and displayed paradoxical expression trends—all serum miRNAs were upregulated, whereas all tissue miRNAs were downregulated. This phenomenon is not isolated but has been similarly documented across various cancer types. For instance, hepatocellular carcinoma is characterized by elevated serum miR-122 levels alongside significantly reduced miR-122 expression in tumor tissues [35,36]. Similarly, in breast cancer, upregulated miR-195 has been observed in whole blood [37], while its expression is decreased in tumor tissues [38]. These findings underscore the widespread inverse relationship between circulating and tissue-resident miRNA expression profiles, aligning with previous reports. Mjelle et al. demonstrated distinct miRNA expression profiles among tissues, serum, and exosomes from the same patient, suggesting that circulating miRNAs are unlikely to be merely passive leakage products from tumor tissues but are probably subject to active regulatory release mechanisms [39]. Existing literature confirms that cells can selectively sort and release nucleic acids and proteins via exosomes, thereby mediating intercellular communication [40]. Integrating our findings with established research, we hypothesize that ovarian cancer cells may selectively sort and release specific miRNAs via exosomal pathways [41]. This mechanism could plausibly explain the observed expression profile—characterized by global upregulation in serum and downregulation in tissues. It is important to note that this study did not perform exosome isolation, characterization, or functional validation experiments. Therefore, the aforementioned mechanistic inference is derived solely from bioinformatics data and existing literature, necessitating further experimental verification.

KEGG pathway enrichment analysis revealed that the target genes of both serum- and tissue-derived miRNAs converged on multiple shared signaling pathways. Based on these findings, we hypothesize a functional convergence between the two miRNA sets: although they regulate distinct target genes, they may exert coordinated biological effects. Specifically, tissue miRNAs appear to govern intrinsic oncogenic signaling within tumor cells, whereas serum miRNAs likely modulate tumor progression through indirect systemic influences. Crucially, both cohorts converge on the core regulatory nodes of the cell cycle and the PI3K-Akt signaling pathway. It is important to emphasize that this inference of functional convergence is a bioinformatics hypothesis derived from enrichment analysis; the precise regulatory architecture requires further experimental dissection.

Although this study preliminarily elucidates the distinct expression profiles and regulatory disparities of serum and tissue miRNAs in early-stage ovarian cancer, several limitations warrant acknowledgment. First, the inherent biases of this retrospective secondary analysis of public databases—compounded by baseline confounding and batch-effect heterogeneity arising from disparate sample sources, sequencing platforms, and preprocessing standards across cohorts—could not be entirely mitigated by ComBat correction. This potentially compromises the robustness of the results and necessitates validation through prospective clinical trials and quantitative reverse transcription PCR (qRT-PCR). Furthermore, the limited sample size of the external validation set (*n* = 34) for the tissue cohort undermines analytical reliability and model generalizability, particularly given the high-dimensional nature of miRNA data and the complexity of the machine learning algorithms employed. Notably, several models—specifically the XGBoost model in the tissue cohort and the Random Forest (RF) and XGBoost models in the serum cohort—exhibited overt overfitting, with training set AUCs approaching 1.0 and significant performance degradation in validation sets. This highlights the need for optimized regularization and expanded sample sizes. Additionally, the absence of critical clinical covariates (e.g., menopausal status, treatment history, histological subtypes) precluded the development of integrated diagnostic models, a limitation that must be addressed in future studies incorporating comprehensive clinical metadata. Finally, all findings—from differential expression screening and model construction to target gene interactions and protein–protein interaction (PPI) network analysis—are derived from in silico predictions, lacking empirical validation through functional assays, dual-luciferase reporter experiments, in vivo models, or independent qRT-PCR of clinical samples. This deficiency in translational evidence underscores the imperative for future prospective multi-center cohorts and foundational wet-lab experiments to substantiate these computational inferences and advance miRNA biomarkers toward clinical utility.

## 4. Methods and Materials

### 4.1. Data Acquisition and Preprocessing

#### 4.1.1. Tissue Samples

The tissue analysis cohort integrated two GEO datasets: GSE169314 (platform GPL25134), initially comprising 87 early-stage ovarian cancer samples, and GSE261800 (platform GPL17303), originally containing 20 ovarian cancer samples paired with 20 normal ovarian controls. Given the inherent data heterogeneity arising from different detection platforms, a unified standardization pipeline combined with ComBat batch-effect correction was applied to mitigate platform-specific and batch-related biases. Following preprocessing, dataset integration, and selection for early-stage cases, the analysis cohort ultimately comprised 87 early-stage ovarian cancer samples and 20 normal controls. For external validation, data from the TCGA-OV project (RNA-Seq, UNC Illumina HiSeq) were integrated with the GSE53829 dataset (platform GPL18138). The TCGA-OV dataset originally contained 576 ovarian cancer samples, while GSE53829 comprised 45 ovarian cancer samples and 14 normal controls. This external cohort encompassed multiple sequencing and microarray platforms, introducing significant technical and biological heterogeneity; therefore, a data harmonization strategy identical to that used for the internal cohort was rigorously implemented. Subsequent to uniform quality control, early-stage case selection, and batch correction, 34 early-stage ovarian cancer samples and 14 normal controls were ultimately retained for external validation.

#### 4.1.2. Serum Samples

The serum analysis cohort was derived from the GSE106817 dataset (platform GPL21263), which initially comprised 4046 female serum samples, including 333 ovarian cancer cases, 66 borderline ovarian tumors, 29 benign ovarian diseases, 2759 non-cancer controls, and 859 patients with other solid tumors. From this dataset, 110 cases of stage I–II early-stage ovarian cancer and 200 non-cancer controls were selected, yielding a total of 310 samples. These were randomly partitioned into a training set (*n* = 217) and an internal validation set (*n* = 93) at a 7:3 ratio. For external validation, the GSE211692 dataset (platform GPL21263) was employed, utilizing a detection platform identical to that of the discovery cohort. This external dataset originally contained 9921 serum samples from various tumor and non-tumor conditions. Strict inclusion and exclusion criteria were uniformly applied, and age-stratified matching was performed between cases and controls to minimize heterogeneity in clinical characteristics. Following stringent early-stage sample screening and age matching, 168 early-stage ovarian cancer cases and 200 non-cancer controls were ultimately retained for external validation.

#### 4.1.3. Data Normalization and Batch Effect Correction

A unified preprocessing pipeline was implemented for the tissue cohorts. Raw logCPM values were converted to counts per million (CPM). Subsequently, expression matrices within each cohort were integrated, and non-expressed miRNAs were filtered out. Quantile normalization, log_2_ transformation, and per-miRNA Z-score standardization were then sequentially performed. To address multi-platform heterogeneity in the tissue discovery cohort, ComBat batch-effect correction was applied to mitigate platform-specific biases and standardize the data baseline, yielding a normalized expression matrix for downstream analyses.

To strictly preclude data leakage, the preprocessing pipeline was executed independently for each cohort as follows:

(1) Training Set: Standardization and Z-score scaling were conducted exclusively on the training set samples. The ComBat correction model was trained and applied herein to derive the standardization parameters and batch-correction model.
(2) Validation/External Validation Sets: These sets were entirely excluded from parameter estimation. Instead, the standardization parameters and ComBat model derived from the training set were directly applied to independently correct both the internal and external validation sets, thereby ensuring no data leakage.

This comprehensive strategy—encompassing quality control, normalization, and batch harmonization—was uniformly applied across internal and external cohorts. It systematically mitigated heterogeneity arising from disparities in detection platforms, experimental procedures, and sample baselines.

### 4.2. Screening of Hub miRNAs by WGCNA Combined with Differential Analysis

All differential expression analyses, co-expression network constructions, and hub miRNA screenings were strictly confined to the respective training sets for tissues and serum; validation sets were entirely excluded from feature selection workflows to preclude data leakage. Initially, differential expression analysis was performed using R software (version 4.4.2) and the R packages limma (v3.62.2), edgeR (v4.4.2), and DESeq2 (v1.46.0), applying thresholds of |log_2_FC| > 0.5 and *p* < 0.05. The intersected miRNAs identified by all three algorithms were retained. To ensure analytical consistency, all subsequent analyses were uniformly based on the differential expression results generated by the limma package. Subsequently, WGCNA was performed on the training set data to construct a co-expression network, and the key module exhibiting the highest correlation with the early-stage ovarian cancer phenotype was identified. Within this module, miRNAs meeting the criteria of |GS (Gene Significance)| > 0.5 and |MM (Module Membership)| > 0.7 were designated as key miRNAs. The intersection of these differentially expressed miRNAs and WGCNA-identified key miRNAs was then subjected to the natural breaks (Jenks) classification method to determine the optimal core miRNA set, which served as the final hub miRNAs for model construction.

### 4.3. Machine Learning Model Construction and External Validation

Modeling analyses were conducted using R (v4.4.2) with pertinent packages, including caret (v7.0.1), glmnet (v4.1.10), xgboost (v1.7.11.1), sva (v3.54.0), and rms (v8.1.0), based on the expression profiles of the identified serum- and tissue-derived hub miRNAs. To ensure reproducibility, the random number generator was initialized using set.seed (123). Tissue samples were subjected to sequential ComBat batch-effect correction followed by Z-score standardization, whereas serum samples underwent Z-score standardization exclusively. To preclude data leakage, all validation sets were processed strictly using preprocessing parameters derived from the training set. To mitigate class imbalance within the training cohort, a combined strategy incorporating the Synthetic Minority Over-sampling Technique (SMOTE), sample weighting, and class weighting was implemented.

Seven machine learning classifiers were constructed: Least Absolute Shrinkage and Selection Operator (LASSO), Linear Discriminant Analysis (LDA), Random Forest (RF), Support Vector Machine (SVM), eXtreme Gradient Boosting (XGBoost), k-Nearest Neighbors (kNN), and Naive Bayes. Hyperparameter optimization was performed via five-fold cross-validation integrated with grid search, with the objective of maximizing the cross-validated Area Under the Curve (AUC). The optimal classification threshold was subsequently determined using Youden’s index. Model training incorporated predefined parameter grids alongside fixed parameters; notably, optimal hyperparameters for LASSO and kNN were selected through cross-validation. Model performance was comprehensively evaluated using AUC, accuracy, sensitivity, specificity, and the F1 score and validated across the training set, internal validation set, and independent external dataset. Principal Component Analysis (PCA) was employed to verify the efficacy of batch correction. Feature contributions within the XGBoost model were interpreted using SHapley Additive exPlanations (SHAP), and visualization analyses were completed using ROC curves, nomograms, and other relevant graphical representations.

### 4.4. Target Gene Prediction and Functional Enrichment Analysis

Target genes of the identified core miRNAs (both tissue- and serum-derived) were predicted using three established databases: TargetScan, miRDB, and miRTarBase. To enhance predictive reliability, only intersecting targets corroborated by all three databases were retained. The STRING database was subsequently utilized to construct protein–protein interaction (PPI) networks for these consensus targets. Networks were visualized using Cytoscape software (v 12.0), and the top 10 hub genes—ranked by degree value—were selected to delineate the interaction landscape. Separate PPI networks were constructed and visualized for targets specific to tissue-derived and serum-derived core miRNAs, respectively.

Subsequently, functional enrichment analyses were performed using the enrichR package (v 3.4) in R, encompassing Gene Ontology (GO) terms—including Biological Process (BP), Cellular Component (CC), and Molecular Function (MF)—and Kyoto Encyclopedia of Genes and Genomes (KEGG) pathways. Pathways and functional categories with a *p* < 0.05 were deemed statistically significant and were identified as key biological processes associated with ovarian cancer.

## 5. Conclusions

Integrated analysis revealed that although core serum and tissue miRNAs in early-stage ovarian cancer exhibited paradoxical expression trends, they converged upon largely consistent primary regulatory pathways. This suggests that serum miRNAs may mirror the molecular landscape of tumor tissues to a certain extent. Based on available data and the literature, we hypothesize that serum miRNA release is not solely attributable to passive tissue leakage but may involve actively regulated secretory processes; however, this inference remains a bioinformatics-derived hypothesis. Nevertheless, diagnostic models constructed using these core miRNAs demonstrated robust stability and favorable discriminatory performance.

In summary, this study posits that serum miRNAs hold considerable potential as non-invasive biomarkers for early ovarian cancer detection, offering valuable insights for future investigations. Constrained by the study design, no empirical laboratory experiments were conducted; consequently, all mechanistic conclusions regarding miRNA secretion patterns and functional associations remain hypothetical. Subsequent validation of these analytical findings and inferences will necessitate further clinical sampling and foundational experimental work.

## Figures and Tables

**Table 1:** Age analysis of patients with early-stage ovarian cancer in serum and tissue cohorts.

Serum Analysis Cohort of Ovarian Cancer Characteristics				
Feature	Overall *N* = 110	Stage I *N* = 80	Stage II *N* = 30	*p*-value
Age (years, Mean ± SD)	53.5 ± 11.8	51.4 ± 11.5	59.1 ± 11.1	0.002
Serum external validation cohort characteristics of ovarian cancer				
Feature	Overall *N* = 168	Stage I *N* = 131	Stage II *N* = 37	*p*-value
Age (years, Mean ± SD)	53.8 ± 12.9	52.6 ± 12.9	57.5 ± 12.3	0.039
Analyze the characteristics of ovarian cancer in the organizational cohort				
Feature	Overall *N* = 87	Stage I *N* = 61	Stage II *N* = 26	*p*-value
Age (years, Mean ± SD)	54.6 ± 13	53.5 ± 13.6	57.3 ± 11.2	0.039
External validation cohort of ovarian cancer characteristics				
Feature	Overall *N* = 34	Stage I *N* = 7	Stage II *N* = 27	*p*-value
Age (years, Mean ± SD)	59.2 ± 11.4	56.4 ± 8.5	59.9 ± 12	0.397

**Table 2:** Details of serum and tissue core miRNAs.

Specific information on serum core miRNAs					
Gene	GS	MM	log_2_FC	padj	regulation
hsa-miR-4783-3p	0.807	0.880	2.794	<0.001	Up
hsa-miR-4532	0.796	0.877	2.414	<0.001	Up
hsa-miR-663a	0.761	0.933	1.497	<0.001	Up
hsa-miR-6802-5p	0.756	0.889	0.906	<0.001	Up
hsa-miR-4258	0.753	0.868	1.682	<0.001	Up
hsa-miR-373-5p	0.746	0.780	1.437	<0.001	Up
hsa-miR-4675	0.745	0.909	1.567	<0.001	Up
hsa-miR-1233-5p	0.739	0.881	1.760	<0.001	Up
Specific information about the core miRNAs of the organization					
Gene	GS	MM	log_2_FC	padj	regulation
hsa-miR-191-5p	−0.999	0.984	−2.563	<0.001	Down
hsa-miR-106b-3p	−0.983	0.977	−2.444	<0.001	Down
hsa-miR-28-3p	−0.979	0.989	−2.538	<0.001	Down
hsa-miR-126-5p	−0.977	0.972	−2.495	<0.001	Down
hsa-miR-92b-3p	−0.970	0.978	−2.535	<0.001	Down
hsa-miR-143-3p	−0.967	0.936	−2.513	<0.001	Down
hsa-miR-423-3p	−0.965	0.975	−2.518	<0.001	Down
hsa-miR-3184-5p	−0.962	0.939	−2.651	<0.001	Down

## Data Availability

The original sequencing data can be downloaded from the GEO and TCGA repositories. The analysis code is available from the corresponding author upon reasonable request.

## Supplementary Materials

The following supporting information can be downloaded at: https://www.mdpi.com/article/10.3390/ijms27125629/s1.
