# Supplementary material for: Comparative Analysis of Serum and Tissue miRNA Expression Profiles and Regulatory Pathways in Early-Stage Ovarian Cancer Using Public Databases
Source: Int J Mol Sci. 2026 Jun 22;27(12):5629. doi: 10.3390/ijms27125629 (PMC13299059; doi:10.3390/ijms27125629)
Supplement: Supplementary file 1 [file ijms-27-05629-s001.zip › ijms-4312397-supplementary.pdf]

Table S1. Performance of Serum and Tissue Cohort Models.

Model	Dataset	AUC	Accuracy	Sensitivity	Specificity	F1
**Serum Cohort Model Performance**						
Model	Dataset	AUC	Accuracy	Sensitivity	Specificity	F1
LASSO	Train	0.973	0.949	0.961	0.943	0.931
LDA	Train	0.976	0.945	0.974	0.929	0.926
RF	Train	1.000	1.000	1.000	1.000	1.000
SVM	Train	0.972	0.954	0.974	0.943	0.938
XGB	Train	1.000	1.000	1.000	1.000	1.000
kNN	Train	0.989	0.949	0.974	0.936	0.932
NB	Train	0.971	0.926	0.974	0.900	0.904
LASSO	Val	0.991	0.979	1.000	0.967	0.971
LDA	Val	0.993	0.979	1.000	0.967	0.971
RF	Val	0.997	0.968	0.970	0.967	0.955
SVM	Val	0.992	0.979	1.000	0.967	0.971
XGB	Val	0.997	0.968	0.970	0.967	0.955
kNN	Val	0.993	0.968	0.970	0.967	0.955
NB	Val	0.994	0.979	1.000	0.967	0.971
XGB	External	0.998	0.984	0.970	0.995	0.982
**Tissue Cohort Model Performance**						
Model	Dataset	AUC	Accuracy	Sensitivity	Specificity	F1
LASSO	Train	0.500	0.508	0.508	0.508	0.508
LDA	Train	0.590	0.615	0.721	0.508	0.652
RF	Train	0.998	0.975	0.984	0.967	0.976
SVM	Train	0.554	0.353	0.574	0.131	0.470
XGB	Train	1.000	1.000	1.000	1.000	1.000
kNN	Train	0.994	0.959	0.984	0.934	0.960
NB	Train	0.707	0.680	0.590	0.771	0.649
LASSO	Val	0.500	0.500	0.500	0.500	0.619
LDA	Val	0.545	0.719	0.769	0.500	0.816

RF	Val	0.865	0.969	1.000	0.833	0.981
SVM	Val	0.539	0.281	0.231	0.500	0.343
XGB	Val	0.859	0.906	0.923	0.833	0.941
kNN	Val	0.958	0.969	1.000	0.833	0.981
NB	Val	0.590	0.875	1.000	0.333	0.929
kNN	External	0.971	0.958	0.941	1.000	0.970
XGB	External	0.758	0.792	0.794	0.786	0.844
